# Patient-Reported Donor Site Quality of Life Outcomes Following Fibula Free Flap Reconstruction

**DOI:** 10.3390/cmtr18010014

**Published:** 2025-02-11

**Authors:** Edgar Ochoa, James Cevallos, Ramon Bustos, Nina Patel, Chase M. Heaton, Rahul Seth, P. Daniel Knott, Andrea M. Park

**Affiliations:** 1 Division of Facial Plastic and Reconstructive Surgery, Department of Otolaryngology-Head and Neck Surgery, University of California-San Francisco, 2320 Sutter Street, Suite 102, San Francisco, CA 94115, USA; 2 Department of Otolaryngology-Head and Neck Surgery, University of California-San Francisco School of Medicine, San Francisco, CA 94143, USA

**Keywords:** head and neck reconstruction, microvascular free flap, quality of life, claw toe deformity

## Abstract

*Study Design:* Retrospective cohort study. *Objective:* To (1) assess post-operative quality of life (QOL) and functional outcomes of fibula free flap (FFF) donor sites, (2) examine the incidence of post-operative claw toe deformities (CTDs) following FFF harvest, and (3) assess the effect of flexor hallicus longus (FHL) muscle preservation on the incidence of post-operative CTDs. *Methods:* Patients aged 18 years or older and at least 6 months from FFF reconstructive surgery completed the Manchester–Oxford Foot Questionnaire (MOXFQ)and the Short-form 36 (SF-36) Questionnaire. The incidence of post-operative CTDs reported by respondents was calculated. We assessed the associations between FHL preservation at time of surgery and QOL and functional outcomes, including the development of post-operative CTDs. *Results:* Sixty patients completed questionnaires at a mean of 38.3 months from surgery. The cohort consisted of 28 patients in whom the FHL muscle and nerve were preserved and 32 patients in whom they were not. Among respondents, 23.3% (14/60) reported post-operative CTDs. FHL status was not associated with post-operative CTDs or with worse scores in the domains of pain (*p* = 0.612), walking/standing (*p* = 0.431), or social functioning (*p* = 0.400). Overall, majority reported high post-operative QOL. *Conclusions:* While majority of patients who undergo FFF reconstruction can expect good long-term donor site QOL and functional outcomes, there are risks of functional impairment, including post-operative CTDs. Preservation of FHL muscle did not affect post-operative QOL outcomes or the incidence of CTDs. These data can inform patient QOL expectations following FFF harvest.

## 1. Introduction

Microvascular free tissue transfer is a reliable method of reconstruction following ablative surgery for cancers of the head and neck [1]. The fibula free flap (FFF) is commonly utilized for oromandibular and midface reconstructions, with excellent functional and aesthetic results [2,3]. 

While detriments to the quality of life (QOL) following FFF reconstruction have been previously reported in the domains of chewing, speech, and appearance, there is a paucity of data regarding long-term patient-reported QOL and functional outcomes at the flap donor site [4,5,6]. Detriments to ankle function and gait, sensory deficits, and chronic pain can develop following FFF harvest [7,8,9,10]. Patients are additionally at risk of developing claw toe deformities, defined by persistent involuntary flexion at the middle joints of the toes. These impairments in toe function are thought to be attributed, at least in part, to flexor hallucis longus (FHL) muscle injury during FFF harvest and subsequent scarring, contraction, and/or atrophy of the FHL muscle [11]. Claw toe deformities may contribute to chronic pain, foot ulceration, and compromised gait and can influence a patient’s recovery and QOL.

Preservation of the tibial nerve and FHL muscle at the time of FFF harvest may offer some protection against the development of claw toe deformities. Although the proximal FHL muscle attachment to the posterior fibula is released during fibula harvest, muscle function may be retained when the tibial nerve is preserved and the muscle attachment is resuspended. Thus, this study aims to (1) assess post-operative functional and QOL outcomes of FFF harvest sites, (2) examine the incidence of self-reported claw toe formation following FFF reconstruction, and (3) assess the association of FHL preservation and resuspension on the incidence of post-operative claw toe deformities. Our findings may be used by surgeons to guide pre-operative planning and inform patient QOL and functional outcome expectations following FFF reconstruction.

## 2. Materials and Methods

This study protocol was approved by the Institutional Review Board of the University of California-San Francisco (UCSF) Medical Center. 

### 2.1. Patient Selection

We reviewed a prospectively maintained database of patients who had undergone head and neck microvascular reconstruction with FFF at the UCSF Medical Center. Patients included in our analysis were aged 18 years or older, at least 6 months from surgery, and able to complete the questionnaires. Patients with incomplete questionnaires and those with post-operative complications at the harvest site were excluded from analysis. All cases of tumor ablation involved malignant lesions; benign lesions were not included within the scope of the study.

### 2.2. Survey Instruments and Data Collection

Patients completed two separate QOL and functional outcomes questionnaires: The Manchester–Oxford Foot Questionnaire (MOXFQ) and the Short-form 36 Questionnaire (SF-36). The MOXFQ is a validated 16-item patient-reported outcome measure for evaluating outcomes of foot or ankle surgery, whereas the SF-36 is a validated self-administered general health questionnaire [12,13]. Upon completion of the questionnaires, patients were additionally asked to report on the development of chronic toe contracture on the side of FFF harvest since time of surgery. No objective measurements to assess reliability of suspension procedure or of post-operative FHL function recovery were conducted as part of this study. 

All survey responses were collected and stored in the institutional Research Electronic Data Capture (REDCap) database, a secure and encrypted electronic platform for observational studies. Patient demographic information and clinicopathologic characteristics were then collected through retrospective review of the medical records. 

### 2.3. Questionnaire Score Interpretation

MOXFQ scores for the domains of pain, walking/standing, and social interaction were tallied on a 5-point Likert scale and then linearly converted into a 0–100 scale. Higher scores reflected worse QOL or functional outcomes. SF-36 questionnaire raw scores for the domains of physical functioning, limitations due to physical health, limitations due to emotional problems, energy/fatigue, emotional well-being, pain, and general health were converted into a range from 0 to 100, with higher scores defining a more favorable health state. Diagnostic criteria for post-operative claw toe deformities involved a positive report of post-operative involuntary ‘toe curling’ on the ipsilateral foot of the fibula donor site by the patient at the time of study questionnaire completion.

### 2.4. Operative Technique

The operative methods utilized in this paper differ only in the preservation and resuspension of FHL during fibula harvest. Both methods first require marking the design of the flap and skin paddle on the lower extremity, which can vary in size depending on anticipated reconstruction needs and patient-specific leg size. The leg is exsanguinated with the use of a tourniquet. A lateral incision is made along the fibula, and a skin paddle is fashioned based on vascular perforators. The flap is elevated with careful dissection of the peroneal artery and vein, and their associated perforators. 

All muscular attachments, including the FHL, are dissected free from the fibula in all patients. The FHL muscle is completely removed from its proximal attachment–the lower posterior surface of the fibula and the adjacent intermuscular septum. Harvest is then completed with upper and lower osteotomies. To minimize post-operative knee and ankle instability, at least 6 centimeters of fibula bone is preserved distally and proximally in all patients. This results in variable lengths of fibula harvested in each patient; however, residual fibula length is largely consistent. 

In FHL preservation and resuspension cases, the FHL musculature, along with the tibial nerve, is left intact to the greatest extent possible during dissection of the pedicle and its branches. This is performed to limit the quantity of functional muscle harvested along with the flap. Following harvest, resuspension of the FHL muscle is completed by securing the proximal FHL muscle—which was previously attached to the distal fibula—to the interosseous membrane and tibialis posterior muscle with the use of vicryl sutures.

Following wound closure, patients are placed in an ankle immobilization cast for 5 days. No lower extremity weight-bearing limitations are imposed, and progressive mobilization is encouraged for all patients immediately post-operation. In addition, all patients are assessed and managed by inpatient physical therapists while hospitalized. 

### 2.5. Analysis

The incidence of post-operative toe contracture reported among respondents was calculated, and statistical analysis was performed to define the association among patients with toe contracture based on FHL preservation status. FHL preservation status was designated based on established surgeon-specific intraoperative techniques for FHL muscle and nerve preservation. Preserved FHL status indicates surgical cases where the FHL muscle was dissected free from the neurovascular sheath and resuspended, and the nerve to the FHL was preserved intraoperatively. For cases where the FHL was not preserved, the FHL muscle and the nerve to the FHL were divided. All FHL preservation surgeries were performed by the same facial plastic surgeon; however, cases without FHL preservation surgeries were conducted by 4 different surgeons within the same department at a single academic medical institution. 

Statistical analyses were performed using two-tailed *t*-tests to compare MOXFQ scores and SF-36 scores among patients with FHL preservation and those without FHL preservation. Univariate logistic regression was utilized to evaluate odds ratios (ORs) with 95% confidence intervals (CIs) for the development of toe contractures. Multivariable logistic regression was performed to evaluate FHL preservation, age, sex, ASA status, smoking status, and follow-up rates. All statistical analyses were performed using R statistical software (v3.6.1). An a priori *p*-value of 0.05 was chosen as the cutoff for statistical significance. 

## 3. Results

After exclusion based on the criteria above, 60 patients consented and completed the MOXFQ and SF-36 questionnaires. Demographic and clinical characteristics of the patient cohort are presented in Table 1. A majority of the patients were aged greater than 60, male, ASA class II, had <10 pack-years smoking history, received adjuvant radiation therapy, and had undergone FFF reconstruction following tumor ablation (76.7%). Additionally, the majority of fibulas were used in the reconstruction of the mandible after mandibulectomy (83.3%). Tourniquet time was significantly longer among patients with FHL not preserved, and follow-up time from surgery until questionnaire response was significantly longer in the FHL preserved group.

### 3.1. Questionnaire Responses

MOXFQ domain means according to FHL preservation status are presented in Table 2. Patients in whom FHL was preserved did not report significantly different scores in the domains of pain (18.6 vs. 21.9, *p* = 0.612), walking/standing (14.0 vs. 18.7, *p* = 0.431), or social functioning (9.6 vs. 13.7, *p* = 0.400). Comprehensive MOXFQ scores were likewise similar between the two groups (14.3 vs. 18.5, *p* = 0.436). 

Table 3 demonstrates SF-36 domain mean scores based on FHL preservation status. Respondents did not report significantly different mean scores for the domains of physical functioning (76.6 vs. 69.2, *p* = 0.261), role limitations due to emotional problems (73.8 vs. 79.2, *p* = 0.570), energy/fatigue (68.8 vs. 69.7, *p* = 0.783), emotional well-being (70.0 vs. 71.0, *p* = 0.788), pain (74.0 vs. 66.6, *p* = 0.246), and general health (71.6 vs. 61.3, *p* = 0.057). Significantly lower mean scores, indicating a less favorable health state, were reported among patients in whom the FHL was not preserved for the domains of role limitations due to physical health (79.5 vs. 0.023, *p* = 0.023) and social functioning (89.7 vs. 73.4, *p* = 0.005).

Among respondents, 23.3% (14/60) reported post-operative claw toe deformities on the side of their surgery. Of these patients, 71.4% (10/14) reported experiencing contracture of the toes “often” or “always”, and 57.1% (8/14) indicated that involuntary toe contracture was “moderately” or “severely” bothersome to their daily lives. In a sub-analysis evaluating patients reporting claw toe deformities, there were no significant differences in rates of self-reported post-operative toe contracture based on FHL preservation status (*p* = 0.214) (Table 4). Additionally, the MOXFQ domain score means for patients with post-operative claw toe deformity status was 24.6, compared to 14.1 reported by their non-claw toe deformity counterparts. Overall, there were no significant differences among MOXFQ domain score means based on post-operative claw toe deformity status. 

### 3.2. Multivariable Analysis 

Table 5 shows a multivariable analysis of possible risk factors for post-operative toe contracture. FHL preservation, age, sex, ASA status, ischemia time, and follow-up >1 year were not associated with post-operative toe contracture. A smoking history of ≥10 pack-years was independently associated with a higher risk of post-operative toe contracture (OR 6.6; *p* = 0.033).

## 4. Discussion

The FFF is frequently utilized after resection of oral cavity or midfacial cancers with osseous infiltration due to its reliability and effectiveness. Understanding patient-reported morbidity of this widely adopted reconstructive option is necessary to optimize long-term post-operative function and QOL. Our study does not capture objective measures of post-operative FHL function recovery in patients based on intraoperative FHL preservation; rather, we aim to assess the QOL impact of toe functioning and how much this affects patient functioning. To the best of our knowledge, this is the largest assessment of patient-reported donor site QOL outcomes in patients undergoing FFF. Our study corroborates previous investigations that have demonstrated satisfactory long-term overall QOL for patients who undergo FFF [14,15,16,17]. Moreover, we specifically demonstrate that function and QOL outcomes focused on the flap donor site are likewise high for the domains of pain, function (walking and standing), and social functioning. These specific patient-reported data may serve to inform pre-operative patient counseling and QOL expectations following FFF harvest.

In this patient cohort, the development of post-operative claw toe deformities was reported by 23.3% of respondents. This rate is in line with previously published reports of this complication following FFF; however, the incidence varies widely among studies [9,18,19]. A majority of those experiencing claw toe indicated symptoms to be frequent and moderately to significantly bothersome, suggesting a significant impact on post-operative QOL in those afflicted. At the time of data collection, however, only one patient had sought evaluation from an orthopedic surgeon to address his/her symptoms, and in this instance, conservative measures involving stretching and taping of the toes in a natural position rather than surgical correction were recommended. Claw toe deformities were present as early as 9.1 months and up to 54.5 months after surgery, likely indicating chronic non-resolving symptomatology for patients. Given the significant risk of toe contracture following FFF, pre-operative counseling of the patient should routinely include disclosure of toe contracture as a potential complication; in addition, routine assessment for toe contracture should be conducted at post-operative follow-up visits, and appropriate referral for physical therapy or to orthopedic providers should be strongly considered depending on the degree of symptomatology. 

Although 23.3% of patients reported post-operative toe contractures, MOXFQ scores were generally indicative of satisfactory long-term post-operative outcomes in the domains of pain, mobility, and ability to carry out social or recreational activities due to foot pain. Though there was a trend toward higher pain scores in patients who reported post-operative toe contracture, this was not statistically significant. An already widely reported risk factor for peri- and post-operative complications, our multivariable analysis showed a smoking history of ≥10 pack-years as an independent risk factor for post-operative toe contracture. Significant smoking history may set the stage for poor tissue perfusion and impaired wound healing and thus may ultimately result in detriments to functional outcomes such as those reported by this cohort.

Although our understanding of which patients will and will not develop CTD remains limited, a 2024 study by Liu et al. assessed the post-operative characteristics of the FHL muscle under ultrasound. Their results showed that in the area of the fibula defect, the FHL muscle displays irregular echo patterns, with blurred or absent muscle texture and no blood flow signals, suggesting ischemia and atrophy in the remaining muscle tissue. These findings reinforce the idea that scarring, muscle contraction, and atrophy occur following flap harvest. The researchers suggested that post-operative ischemic contracture of the muscle is an inevitable outcome, largely due to the necessary ligation of the peroneal artery during harvest, which is the primary blood supply to the FHL muscle [18]. This may indicate that FHL muscle dysfunction and, therefore, toe-curling dysfunction, should be expected following fibula free flap harvest in spite of attempts at preservation or resuspension of the FHL. 

Our analysis did not indicate an association between intraoperative FHL preservation and the development of claw toe deformities, nor worse donor site functional and QOL outcomes. While prior studies have demonstrated decreased FHL strength and range of motion of plantar flexion following FFF harvest to be common, the distinction of FHL preservation status has not appeared to impact functional outcomes [19,20]. In a 2014 cross-sectional cohort study, Van Den Heuvel et al. demonstrated no significant differences in hallux flexion range of motion when comparing osteo-cutaneous flaps (without inclusion of FHL muscle) and osteo-myo-cutaneous flaps (with inclusion of FHL muscle); thus, while the FHL is intimately involved in the flexion of the hallux, removing the FHL may not significantly impact the rate of post-operative toe contractures. 

Apart from a potential impact on QOL outcomes, the decision to harvest FHL with the flap may be informed by the need for additional flap volume, preference for the use of the FHL as a tissue bed for oral mucosa formation, the potential to simplify flap harvesting operation, and its impacts on harvest times. In 2018, Ni et al. demonstrated shorter harvest times when the FHL was harvested with the flap [19]. On the contrary, our study found that longer harvest times were associated with flaps in which the FHL was not preserved. This difference in harvest times may be attributed to surgeon experience—in our study, FHL preservation was routinely performed by a senior surgeon.

We acknowledge the limitations of this study, which include the cohort size, the retrospective nature of the analysis, possible response bias, the inability to account for case-by-case variations in the degree of intraoperative FHL preserved or harvested, and the large degree of variability in the post-operative timing of quality of life survey distribution. Furthermore, the single time-point evaluation of patient responses limits our ability to evaluate changes in patient-reported symptoms from a baseline pre-operative measure or at subsequent time-points post-operation. In addition, we acknowledge that associations of FHL status with overall QOL may be limited without controlling for the multitude of other factors that may contribute to overall QOL.

## 5. Conclusions

While a majority of patients who undergo FFF reconstruction can expect good long-term donor site QOL and functional outcomes, there are risks of functional impairment, including claw toe deformities. Therefore, claw toe deformity should be assessed in the post-operative setting. If this deformity is present and bothersome, patients should be referred to an orthopedic surgeon for evaluation and intervention. In this cohort study, preservation of FHL did not affect patient-reported post-operative QOL and functional outcomes. These data can inform patient QOL expectations following FFF harvest for head and neck reconstruction.

## Figures and Tables

**Table 1:** Demographic and clinical characteristics based on FHL preservation status.

	Total	FHL Preserved	FHL Not Preserved	*p*
n	60	28	32	
Age, years, mean	61.2	59.1	63	0.319
Sex				
Female	24	8	16	0.117
Male	36	20	16	
ASA				
I	4	2	2	0.903
II	34	15	19	
III	22	11	11	
Smoking status				
<10 pack-years	44	21	23	1.000
≥10 pack-years	16	7	9	
Surgical Indication				
Tumor	46	20	26	0.624
ORN	11	6	5	
Other	3	2	1	
Reconstruction Site				
Mandible	50	21	29	0.105
Maxilla	10	7	1	
Skin Paddle Length, centimeters, mean	12.3	11.1	13.3	0.078
Tourniquet Time, minutes, mean	90.9	75.2	104.7	<0.01
Adjuvant Therapy	36	18	22	0.146
Follow-up, months, mean	38.3	49.1	28.6	0.012

**Table 2:** MOXFQ domain mean scores based on FHL preservation status. Higher scores denote worse QOL and functional impairment.

MOXFQ Questionnaire Domain	FHL Preserved, Mean Score	FHL Not Preserved, Mean Score	*p*
Pain	18.6	21.9	0.6121
Walking/Standing	14	18.7	0.4308
Social Functioning	9.6	13.7	0.4002
MOXFQ Comprehensive Score	14.3	18.5	0.4355

**Table 3:** SF-36 domain mean scores based on FHL preservation status. Higher scores denote a more favorable health state.

SF-36 Questionnaire Domain	FHL Preserved, Mean Score	FHL Not Preserved, Mean Score	*p*
Physical Functioning	76.6	69.2	0.261
Role Limitations Due to Physical Health	79.5	57.0	0.023
Role Limitations Due to Emotional Problems	73.8	79.2	0.570
Energy/Fatigue	68.8	69.7	0.783
Emotional Well-being	70.0	71.0	0.788
Social Functioning	89.7	73.4	0.005
Pain	74.0	66.6	0.246
General Health	71.6	61.3	0.057

**Table 4:** FHL preservation status and incidence of toe contracture.

FHL Status	Toe Contracture	No Toe Contracture	*p*
Preserved	4	24	0.2135
Not Preserved	10	22	

**Table 5:** *Multivariable Analysis:* A logistic regression model was used to calculate adjusted odds ratios (ORs) and 95% confidence intervals (CIs) of developing post-operative toe contracture following fibula free flap harvest.

		Univariate			Multivariable		
	n	OR of Post-Operative Toe Contracture	95% CI	*p* Value	OR of Post-Operative Toe Contracture	95% CI	*p* Value
FHL preservation							
No	32	Ref.	Ref.	Ref.	Ref.	Ref.	Ref.
Yes	28	0.37	(0.10–1.34)	0.129	0.58	(0.07–3.99)	0.586
Age, years, mean							
<60	23	Ref.	Ref.	Ref.	Ref.	Ref.	Ref.
≥60	37	1.88	(0.56–6.29)	0.309	0.36	(0.05–2.04)	0.273
Sex							
Female	24	Ref.	Ref.	Ref.	Ref.	Ref.	Ref.
Male	36	0.40	(0.12–1.35)	0.141	0.49	(0.09 – 2.62)	0.400
ASA							
I/II	38	Ref.	Ref.	Ref.	Ref.	Ref.	Ref.
III	22	0.22	(0.04–1.08)	0.062	0.14	(0.01–1.25)	0.122
Smoking status							
<10 pack-years	44	Ref.	Ref.	Ref.	Ref.	Ref.	Ref.
≥10 pack-years	16	2.70	(0.76–9.60)	0.125	6.59	(1.24–43.78)	0.033
Lower limb ischemia time							
<90 min	32	Ref.	Ref.	Ref.	Ref.	Ref.	Ref.
>90 min	28	0.99	(0.24–0.99)	0.991	0.81	(0.12–5.18)	0.825
Follow-up							
<1 year	13	Ref.	Ref.	Ref.	Ref.	Ref.	Ref.
>1 year	44	0.61	(0.15–2.39)	0.477	0.43	(0.06–2.90)	0.367

## Data Availability

Please contact corresponding author.
